# Guanine Oxidation in Double-stranded DNA by MnTMPyP/KHSO_5_: At Least Three Independent Reaction Pathways

**DOI:** 10.1155/MBD.2001.47

**Published:** 2001

**Authors:** Andrea Lapi, Geneviève Pratviel, Bernard Meunier

**Affiliations:** Laboratoire de Chimie de Coordination du CNRS 205 route de Narbonne Toulouse Cedex 4 F-31077 France

## Abstract

In order to better define the mechanism and the products of guanine oxidation within DNA, we investigated the details of the mechanism of guanine oxidation by a metalloporphyrin, Mn-TMPyP, associated to KHSO_5_ on oligonucleotides. We found that the three major products of guanine oxidation are formed by independent reaction routes. The oxidized guanidinohydantoin (**1**) and the proposed spiro compound **3** derivatives are not precursors of imidazolone lesion (**Iz**). These guanine lesions as well as their degradation products, may account for non-detected guanine oxidation products on oxidatively damaged DNA.


### GUANINE OXIDATION IN DOUBLE-STRANDED DNA BY MnTMPyP/$KHSO_5$: AT LEAST THREE INDEPENDENT REACTION PATHWAYS

Andrea Lapi, Geneviève Pratviel and Bernard Meunier*

Laboratoire de Chimie de Coordination du CNRS, 205 route de Narbonne, F-31077 Toulouse Cedex 4, France <bmeunier@lcc-toulouse.fr>

**ABSTRACT**
In order to better define the mechanism and the products of guanine oxidation within DNA, we investigated the details of the mechanism of guanine oxidation by a metalloporphyrin, Mn-TMPyP, associated to $KHSO_5$ on oligonucleotides. We found that the three major products of guanine oxidation are formed by independent reaction routes. The oxidized guanidinohydantoin (**1**) and the proposed spiro compound **3** derivatives are not precursors of imidazolone lesion (**Iz**). These guanine lesions as well as their degradation products, may account for non-detected guanine oxidation products on oxidatively damaged DNA.

**INTRODUCTION**

DNA is a critical cellular target for several oxidation reactions which are associated with aerobic cellular metabolism or to exposure to physical and chemical agents. Oxidative DNA damage plays a key role in mutagenesis, carcinogenesis and cellular aging. Reaction of hydroxyl and alkoxyl radicals, singlet oxygen as well as one-electron oxidants were shown to be responsible for DNA damage.[1,2] Among the four nucleobases, guanine is the preferred target because of its high electronic density and its lowest oxidation potential.[3] However, the nature of the oxidation products and the mechanism of their formation is not well established. One of the most common oxidative modification at guanines is 8-oxo-7,8-dihydro-guanine (8-oxo-G) which is detected in several oxidized DNAs.[4,5] The formation of 8-oxo-G by the attack of reactive oxygen species like OH or $^1O_2$ is well documented.[4-6] However, its formation in the case of electron tranfer oxidation may represent a minor reaction pathway.[7-10] A second well-known product of guanine oxidation is imidazolone (**Iz**)[11-13] associated to its hydrolysis product oxazolone (**Z**)[11]. This product appears as the major one under electron transfer oxidation conditions on oligonucleotides.[7,9,10] A rigorous quantification of **Iz** and 8-oxo-G was undertaken in the case of oxidation of calf thymus DNA with riboflavin known to be mainly a type I photosensitizer (a one-electron oxidant). The amount of these two oxidation products was inferior to the quantity of the total oxidized guanine.[9] In the case of oligonucleotides this difference was even higher.[9] This data implies that some products of guanine oxidation, awaiting identification, may be of significant importance. Because of its low oxidation potential (lower than the one of G)[14,15], 8-oxo-G was postulated to be a transient intermediate in the mechanism of guanine oxidation through electron transfer.[16-18] Thus, some oàther products resulting from 8-oxo-G oxidation may account for the observed missing material. The oxidation of 8-oxo-G derivatives (in the case of an electron transfer mechanism) led indeed to the characterization of new oxidation products, namely guanidinohydantoin, a spiroiminohydantoin and oxaluric acid derivatives.[16-18]

However, 8-oxo-G is not a necessary intermediate in the reaction of guanine oxidation since we reported that the cationic manganese porphyrin (Mn-TMPyP)[19] associated to an oxygen atom donor ($KHSO_5$) was able to perform the quantitative transformation of 2'-deoxyguanosine into imidazolone nucleoside (**dIz**) without intermediate formation of 8-oxo-G.[20] The oxidation of guanine in double-stranded (ds) oligonucleotides (ODNs) by the same system, Mn-TMPyP/$KHSO_5$,[21] did not lead to **Iz** only, but to three main products, a dehydro-guanidinohydantoin (**1**) that was the major product of guanine oxidation in this case, imidazolone (**Iz**) and a proposed 5,8-dihydroxy-7,8-dihydroguanine (**2**) (Figure 1). No 8-oxo-G was detected but new oxidation products. These new guanine oxidation products could therefore be considered as good candidates for the missing material previously observed in guanine oxidation on DNA. Two of the guanine oxidation products, the imidazolone derivative (**Iz**) and the new compound, oxidized guanidinohydantoin (**1**) derivative, were characterized by NMR spectroscopy through the oxidation of a dinucleoside monophosphate model, d(GpT), by Mn-TMPyP/$KHSO_5$.[22] The new compound proposed to be 5,8-dihydroxy-7,8-dihydroguanine (**2**) on the basis of ESI-MS data has not been further characterised up to now. From literature data, the structure **2** may be unstable and the compound may rather correspond to a spiro derivative with the same molecular mass (**3**)[16, 23-25] (Figure 1).

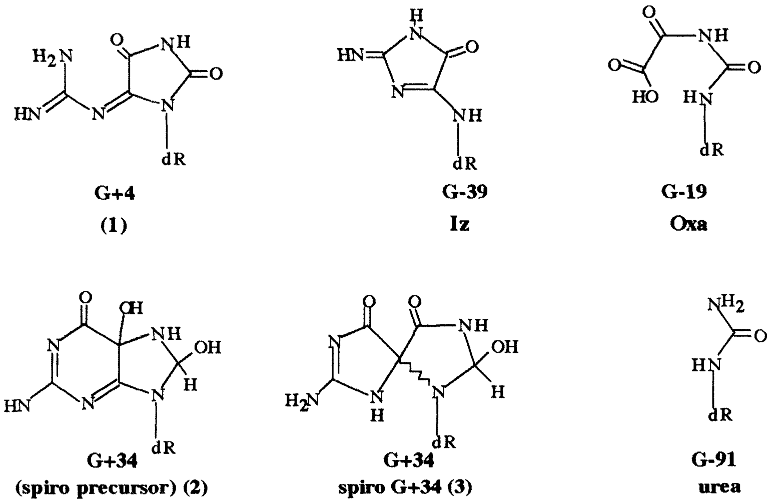

Figure 1: Structure of the products of guanine oxidation by electron transfer mechanism. Their molecular mass is indicated with respect to that of guanine. dR stands for 2-deoxyribose or an intrastrand linkage.

In order to better define the mechanism and the products of guanine oxidation, we investigated the details of the mechanism of guanine oxidation by Mn-TMPyP/$KHSO_5$ on oligonucleotides. We found that the three observed products of guanine oxidation in ds DNA are formed by independent reaction routes. The oxidized guanidinohydantoin (**1**) and the proposed compound **3** derivatives are not precursors of imidazolone lesion (**Iz**). We also studied their stabilities. The oxidation products as well as their degradation products do not show high UV absorption coefficients. They, or their degradation products, may account for non-detected guanine oxidation products on oxidatively damaged DNA.

### MATERIALS AND METHODS

*Materials.* Potassium monopersulfate, $KHSO_5$ (triple salt 2 $KHSO_5$. $K_2SO_4$. $KHSO_4$, Curox®) was from Interox. Oligonucleotides were synthesized by standard solid-phase β-cyanoethyl phosphoramidite chemistry. They were purified by HPLC using a semi-preparative reverse-phase column (Nucleosil C18, 10 µm from Interchrom, Montluçon, France); eluents, A = 0.1 M triethylammonium acetate (TEAA) (pH 6.5), B = $CH_3CN$; linear gradient, 1% to 20% B over 60 min; flow rate, 2.5 mL/min; λ = 260 nm). Mn-TMPyP was prepared as previously described.[26] Labeled water (96.5 atom % $^{18}O$) was from Euriso-top (Saclay, France).

*Oxidation of ODN I and ODN II by Mn-TMPyP/$KHSO_5$.* A typical experiment consisted of preincubation of ODN (10 µM duplex) with Mn-TMPyP (10 µM) in Tris/HCl buffer pH 7 (50 mM), NaCl (100 mM) at 0 °C. The reaction was initiated by the addition of $KHSO_5$ at a final concentration of 500 µM. Final volume was 100 µL. After 5 min at 0 °C, the reaction was stopped by addition of Hepes buffer pH 8 (10 mM) and then directly injected for HPLC or HPLC/ESI-MS analysis. The whole reaction medium (100 µL) was injected. Concentrations in brackets are final concentrations. In the case of the oxidation of ss ODN IIa and ss ODN IIb a concentration of 20 µM of oligonucleotide was used. For the product stability study the reaction medium, once quenched by the addition of Hepes buffer, was allowed to stay at 0 °C for 2 h and then analyzed by HPLC/ESI-MS.

*Labeling experiments.* The reaction was performed as described above, except that the reactants were dissolved in $H_2{}^{18}O$. The reaction mixture was directly injected for HPLC/ESI-MS analysis.

*Investigation of the isotopic pattern of **Iz** and **1** in oligonucleotides oxidation in $H_2{}^{18}O$.* The oxidation of ds ODN IIa/IIb and ODN IIa in Tris/HCl buffer was carried out as described above using a $KHSO_5$ concentration of 100 µM or 500 µM. In ammonium acetate buffer (pH 7, 100 mM) the oxidant concentration was 100 µM while in phosphate buffer (pH 7, 100 mM), sodium acetate (pH 7, 100 mM) and trietylammonium acetate buffer (pH 7, 100 mM) the concentration of $KHSO_5$ was 50 µM. The reaction mixture was analyzed by HPLC/ESI-MS focalizing the mass detector on the $[M-2H]^-$ signals of **Iz** and **1** containing oligonucleotides. The instrument sensibility was set to 0.042 amu step. In order to compare the products' isotopic patterns with the ones of the $^{16}O$ containing isotopes, the same analysis was carried out for the oxidation of ODN IIa/IIb in $H_2{}^{16}O$ under the standard condition reported above.

*HLPC-ESI/MS analyses.* The reaction mixtures (100 µL) were analyzed by HPLC, with an analytical reverse phase column (Uptisphere 5 µm HDO, 250 x 4.6 mm, from Interchrom, Montluçon, France) eluted with a linear gradient (eluents, A = 10 mM TEAA pH 6.5, B = $CH_3CN$; 1-10% B over 60

min; flow rate, 1 mL/min; λ = 260 nm). A diode array detector (Waters) allowed detection of the products at 260 nm and monitoring of UV-vis spectra. For HPLC/ESI-MS analysis the column was coupled to an electrospray mass spectrometer, a Perkin-Elmer SCIEX API 365 equipped with a turbo ion spray source. The temperature of the gaz ($N_2$) stream was set at 450 °C. The flow eluted from the column (50% volume) was introduced into the electrospray source. The analysis were carried out in the negative mode. The use of the turbo ion spray source allowed us to perform HPLC/ESI-MS analyses from diluted duplex oligonucleotides solutions (100 µL at a concentration of 10 µM = 1 nmole of duplex injected).

*Purification of 1 and 3.* Olidonucleotides carrying lesions **1** and spiro **G+34** (**3**), from the oxidation of ODN I, were purified by HPLC with an analytical reverse phase column (Uptisphere 5 µm HDO, 250 x 4.6 mm from Interchrom, Montluçon, France) eluted with a linear gradient (eluents, A = 0.1 M TEAA pH 6.5, B = $CH_3CN$; 1-10% B over 60 min; flow rate, 1 mL/min; λ = 260 nm). The collected fractions were liophylized.

*Stability of lesions 1 and 3.* ODN I was oxidized on a two fold scale compared to the typical reaction conditions described above. The collected fractions of oxidized strand containing one lesion **1** or one lesion **3** were lyophilized and then dissolved in 200 µL of water separately. The purity of the collected product was analyzed by HPLC/ESI-MS on half of the solution (100 µL) while the other 100 µL sample was heated at 90 °C for 15 min and then analyzed by HPLC/ESI-MS.

*Evolution of lesion 3 in water and pH 8.8.* The oligonucleotide strand containing the spiro **G+34** lesion **3** was collected from a 10 fold scale oxidation of ODN I. After the lyophilization procedure, half of the material was recovered in water (320 µL) while the other half in 320 µL of Tris/HCl buffer (pH 8.8, 100 mM). Each solution was then divided in four aliquots. One of them was analyzed by HPLC immediately while the other three were heated at 90 °C in a water bath for 15, 30 and 60 min respectively after which they were analyzed by HPLC. In the same manner, the stability of purified spiro **G+34** was monitored in Tris/HCl buffer (pH 7, 50 mM). In a separate experiment an aliquot of spiro **G+34** was heated in water at 90 °C for 60 min. After which Tris buffer at pH 8.8 (final concentration 100 mM) was added and the mixture was heated at 90 °C for 15 min and analyzed by HPLC.

### RESULTS AND DISCUSSION

We previously reported that the DNA degradation mediated by Mn-TMPyP/$KHSO_5$ within G-rich ds ODNs, were due to chemical modifications of guanines.[21] The Mn-TMPyP/$KHSO_5$ system used for the oxidation of double-stranded G-rich oligonucleotides has the advantage of being a pure electron abstracting agent (no traces of OH nor $^1O_2$ were formed during the reaction). The mechanism was shown not to be a "one-electron" tranfer mechanism but a "two-electron" oxidation of guanine with the intervening of a guanine-cation $(G\text{-}H)^+$ instead of a guanine radical (G-H) .[20] Labeling experiments in $H_2{}^{18}O$ allowed us to propose a mechanism of guanine oxidation which involved the formation of **2** by trapping of the guanine cation at C5 by a molecule of water followed by the attack of another molecule of water at C8. This product was then proposed to be oxidized into **1** to give **Iz**.[21] (see Figure 1 for structures). Compound **1** was also proposed to result from the attack of $HSO_5^-$ at C5 of the guanine cation.[20] In the first part of this work we investigated guanine oxidation by Mn-TMPyP/$KHSO_5$ on two different oligonucleotide sequences in order to verify that the product distribution was not sequence dependent. However, the single- over double-stranded structure of DNA was found to influence the distribution of the guanine oxidation products. In the second part, the $^{18}O$-labeling of the products was carefully measured to confirm the competition between $H_2O$ and $HSO_5^-$ as trapping nucleophiles on the route of the formation of the oxidation products. Finally, the stability of the oxidation products was studied.

### 1- Oxidation of guanine in single- and double stranded oligonucleotides by Mn-TMPyP/$KHSO_5$.

The G-rich double-stranded duplexes selected in this work consisted of the self complementary 5'-CAGCTG (ODN I) for the first one and 5'-CAGGTG (ODN IIa) hybridized with 5'-CACCTG (ODN IIb) for the second one. The oxidation reactions were carried out in 50 mM Tris/HCl buffer pH 7, 100 mM NaCl, at 0 °C for 5 min using a ODN (duplex)/Mn-TMPyP/$KHSO_5$ ratio of 1:1:50, the concentration of the duplex ODN was 10 µM. The analysis was performed using reverse phase HPLC coupled with negative electrospray mass spectrometry (ESI-MS). The concentrations of the reactants were significantly lower than the ones used previously[21] due to the on-line turbo ion spray source that increased the detection sensitivity. The liquid chromatography allowed the sodium counter ions of the ODNs, that were reacted in Tris/HCl buffer, to exchange with the triethylammonium cations of the mobile phase.

*Oxidation of the ODN IIa/ODN IIb duplex structure.* The chromatographic analysis separated the two strands of the duplex, ODN IIa eluted at 53.4 min, ODN IIb at 63.4 min. The oxidized strands eluted before their corresponding non-modified strand at retention times ranging from 46.9 to 48.7 min for ODN IIa and from 55.5 to 57.9 min for ODN IIb. The two oligonucleotides showed a similar conversion (50 to 70%). The $[M\text{-}2H]^{2-}$ region of the on-line ESI-MS spectra of the different HPLC peaks exhibits the highest intensity of $[M\text{-}nH]^{n-}$ signals for all mass analyses under the experimental conditions used. The results are summarized in Table I. The $[M\text{-}2H]^{2-}$ signal of the non modified ODN IIa was at *m/z* 914.9. Three peaks of oxidized strands of DNA were separated. Under the HPLC peak at 46.9 min, the major $[M-2H]^{2-}$signal at *m/z* 932.2 corresponded to a product of +34 amu compared to the non-modified ODN IIa. It was attributed previously to structure **2**[21] but may rather correspond to structure **3** (see Section 4). This lesion will be now named the spiro **G+34** lesion. Under the same HPLC peak, a signal at *m/z* 848.1 indicating a product with a loss of -133 amu from the initial ODN IIa, may correspond to an abasic site generated by the release of a guanine (or a guaninine oxidation product) by hydrolysis of the glycosidic bond.

The major HPLC peak at 47.7 min (Figure 2B) contains four products of ODN IIa oxidation (i) the dehydro-guanidinohydantohin (**1**) (*m/z* 917.0, G+4 amu) (ii) imidazolone (**Iz**) (*m/z* 895.4, G-39 amu) (iii) a $[M-2H]^{2-}$ signal at *m/z* 839.3 (-152 amu) may be due to the release of an oxidized guanine residue induced by a β-elimination at 2' during ESI-MS[26] (iv) a signal at *m/z* 982.6, corresponding to an unknown product with an increase of molecular mass of +135 amu compared to the undamaged oligonucleotide, was referred to as being the **G+135** lesion.

A $[M-2H]^{2-}$ signal (*m/z* = 982.7) is the major species present in the HPLC peak at 48.7 min. Two lesions with the same **G+135** mass were found at different Rt for ODN IIa (47.7 and 48.7 min).

The same reaction products are also observed in the oxidized ODN IIb massif: compound **G+34** (Rt = 55.5 min, *m/z* = 892.1), imidazolone modification (**Iz**) (Rt 56.1 min, *m/z* = 855.2), **1** (Rt 56.1 min, *m/z* = 876.8) and the two **G+135** products (Rt = 57.0 and 57.9, *m/z* = 942.7).

*Oxidation of ODN IIa and ODN IIb single-strands.* The HPLC profiles of separated oxidation reactions showed the same oxidation products, except the absence of the peak corresponding to compound **G+34** (Rt = 46.9 for ODN IIa and 55.5 for ODN IIb), for both ss ODNs. This data was confirmed by the HPLC/ESI-MS analysis. The formation of the spiro **G+34** lesion was thus dependent on a double-stranded structure of the DNA target.

One remark, in ODN IIb, we observed two oxidation products eluting at different Rt (56.1 and 57.0), both corresponding to a loss of molecular mass of -39 amu, attributed to an imidazolone-containing oligonucleotide (Table I). The fact that ODN IIb carries only one guanine suggests that the two products are not due to two different positionning of the same lesion within the sequence. One hypothesis might be that these two oxidation products might correspond to α- or **β-dIz** nucleoside units within the oligonucleotide.[27]

**Table I.** Selected HPLC/ESI-MS data of oxidized products of ds ODN IIa/IIb.

| *m/z* | | | | | Proposed structure | |
|---|---|---|---|---|---|---|
| Obs.[a] | Calc. | ΔM[b] | Rt (min) | Intensity (%)[c] | for G lesion | *m/z* ($H_2{}^{18}O$)[d] |
| 914.9 | 915.1 | | 53.4 | 20.5 | ODN IIa | 914.8 |
| 848.1 | 848.6 | -133 | 46.9 | 4 | abasic site | 848.4 |
| 932.1 | 932.1 | 34 | | | **3** | 932.9[e] |
| 917.0 | 917.1 | 4 | 47.7 | 14.3 | **1** | 917.9 |
| 895.4 | 895.6 | -39 | | | **Iz** | 895.4 |
| 982.6 | | 135 | | | **G+135** | 983.5 |
| 839.3 | | -152 | | | fragmentation (?) | |
| 982.7 | | 135 | 48.7 | 2.5 | **G+135** | 983.4 |
| 874.8 | 875.1 | | 63.4 | 30 | ODN IIb | 874.9 |
| 892.1 | 892.1 | 34 | 55.5 | 1.8 | **3** | 892.7[e] |
| 876.8 | 877.1 | 4 | 56.1 | 15 | **1** | 877.8 |
| 855.3 | 855.6 | -39 | | | **Iz** | 855.3 |
| 855.2 | 855.6 | -39 | 57.0 | 7.5 | **Iz** | 855.3 |
| 808.1 | 808.6 | -133 | | | abasic site | 808.4 |
| 942.7 | | 135 | | | **G+135** | 943.4 |
| 942.5 | | 135 | 57.9 | 4.3 | **G+135** | 943.6 |

a) mean value from 3 experiments
b) ΔM : mass of the modified ODN minus mass of the initial ODN.
c) based on the area of the HPLC peak (UV detection 260 nm)
d) major peak of isotopic distribution.
e) increase of 2 amu compared to *m/z* in $H_2{}^{16}O$ if analysis is performed without HPLC separation. Exchange of one $^{18}O$ during chromatography.

*Oxidation of the ODN I duplex structure.* Identical guanine oxidation products were found on the second duplex ODN IIa /ODN IIb compared to the self-complementary 5'-CAGCTG (ODN I) duplex previously studied.[21] The results of the HPLC/ESI-MS analysis of the oxidation of ODN I are reported on Table II under the new experimental conditions used in this work. The major lesion consisted of a product with a molecular mass increase of +4 amu, dehydro-guanidinohydantoin (**1**) compared to the initial mass of guanine, another product with an increase of mass of + 34 amu (**3**) and a product corresponding to the formation of imidazolone residue (**Iz**). Under the new diluted conditions used in this work, the **G+135** lesion was also observed as two separated peaks on ODN I.[21]

Table II. Selected HPLC/ESI-MS data of oxidized products of ds ODN I.

| *m/z* | | | | | Proposed structure |
|---|---|---|---|---|---|
| Obs. | Calc. | ΔM[a] | Rt (min) | Intensity (%) | for G lesion |
| 894.8 | 895.1 | | 55.9 | 36 | ODN I |
| 911.9 | 912.1 | 34 | 49.2 | 12 | **3** |
| 896.9 | 897.1 | 4 | 49.9 | 26.5 | **1** |
| 875.4 | 875.6 | -39 | | | **Iz** |
| 875.3 | 875.6 | -39 | 50.7 | 14.5 | **Iz** |
| 962.4 | | 135 | | | **G+135** |
| 962.6 | | 135 | 51.5 | 11 | **G+135** |

a) ΔM : mass of the modified ODN minus the mass of the initial ODN.
b) based on the area of the HPLC peak (UV detection 260 nm)

In conclusion, in a reproducible manner, the oxidation of guanines within ds and ss oligonucleotides by Mn-TMPyP/$KHSO_5$, at neutral pH and at 0 °C, leads to dehydro-guanidinohydantoin derivative **1** as the main product associated to a lower amount of imidazolone lesion **Iz** in all the sequences tested. Modified ODNs carrying one spiro **G+34** lesion are only observed in the case of the oxidation of ds ODNs. A fourth type of DNA damage (unknown structure) appears as a lesion with a **G+ 135** amu increase of mass compared to the mass of the initial ODN. This lesion was observed on single- and double-stranded oxidized DNAs. It must be noted that this lesion was not detected by us before in studies performed under different oxidation conditions.[21]

**2- Competition between $KHSO_5$ and $H_2O$ as trapping reagent for a common guanine cation intermediate.** On the basis of previous $^{18}O$ labeling studies on the mechanism of the oxydation of guanine on nucleosides[20] and oligonucleotides[21] by MnTMPyP/$KHSO_5$, we proposed that an intermediate guanine cation at C5 of G was the initial event of guanine oxidation. This intermediate would undergo two competitive reaction pathways: it could be trapped by the nucleophylic attack of a molecule of water or a $HSO_5^-$ anion. We observed that **Iz** did not show any $^{18}O$ incorporation from labeled water on the nucleoside model.[20] This implied an exclusive nucleophilic attack of $HSO_5^-$ at C5 of G and can be explained by the peroxide being a better nucleophile than water. $KHSO_5$ does not exchange oxygen atoms with water. On the opposite, within ds ODNs, **Iz** that had incorporated one $^{18}O$, from labeled water, was the major product. We proposed that the attack of the $HSO_5^-$ anion on the guanine cation, in a double-stranded ODNs, was reduced by the fact that the negatively charged peroxide has a restricted access to the polyanionic DNA.[21] We also know, concerning the products formed during the reaction, that **Iz** does not exchange its oxygen atom with solvent[12] and that compound **G+34** exchanges one of its two oxygen atoms with water.[21] For compound **1**, the exchange is not known. To confirm the $KHSO_5/H_2O$ competition hypothesis, we carried out oxidation of duplex ODN IIa /IIb and ss ODN IIa in labeled water ($H_2{}^{18}O$, 96.5 atom % labeled) with two concentrations of $KHSO_5$. The guanines located on single strands should be more accessible to $KHSO_5$ than on double-stranded duplexes. Moreover the lowering of the concentration of $KHSO_5$ should favor $H_2O$ attack. The results of the oxidation of ds ODN IIa/ODN IIb in $H_2{}^{18}O$ are reported in Table I and Table III. Two sets of analyses were performed. Table I shows the results of labeling studies under standard conditions of mass analyses. Only the major *m/z* peak of the isotopic distribution was recorded. The quantitative relative abundance of the different peaks of the isotopic distributions is shown on Table III. This evaluated the competition between $KHSO_5$ and $H_2O$ as trapping agents of the guanine cation. A higher resolution for the mass spectrometry record (0.042 amu steps) was used to obtain a clear isotope pattern for each product. In order to increase the mass detector resolution, we focused the collection of the data on two oxidized products, **Iz** and **1** containing oligonucleotides.

Table III. $^{18}O$ incorporation in **Iz** and **1** in the oxidation of ds ODN IIa/IIb and ss ODN IIa in $H_2{}^{18}O$ in 50 mM Tris/HCl buffer pH 7, NaCl 100 mM ($H_2{}^{18}O$).

| | | $^{18}O$ incorporation (%)[a] | | | | | |
|---|---|---|---|---|---|---|---|
| | | ds ODN IIa/IIb | | | ss ODN IIa | | |
| [$KHSO_5$] | Product | no $^{18}O$ | 1 $^{18}O$ | 2 $^{18}O$ | no $^{18}O$ | 1 $^{18}O$ | 2 $^{18}O$ |
| 500 μM | **1** (IIa) | 40 | 50 | 10 | 60 | 40 | - |
| | **1** (IIb) | 40 | 50 | 10 | | | |
| | **Iz** (IIa) | 100 | - | | 100 | - | |
| | **Iz** (IIb) | 100 | - | | | | |
| 100 μM | **1** (IIa) | - | 60 | 40 | 15 | 40 | 45 |
| | **1** (IIb) | - | 50 | 50 | | | |
| | **Iz** (IIa) | 100 | - | | 100 | - | |
| | **Iz** (IIb) | 100 | - | | | | |

a) The values are estimated on the basis of products isotopic patterns.

The results of Table I show that, as expected, the $[M-2H]^{2-}$ signals of the starting ODN IIa and ODN IIb did not change, remaining at *m/z* 914.8 and 875.0, respectively, when the reaction was carried out in labeled water. The $[M-2H]^{2-}$ signals of oligonucleotides containing a spiro **G+34** lesion appeared at *m/z* 932.9 for ODN IIa and 892.6 for ODN IIb in $H_2{}^{18}O$, showing only one $^{18}O$ atom incorporation. However, by direct introduction of the sample in the mass spectrometer, spiro **G+34** showed an incorporation of two $^{18}O$ atoms, one of them exchanged with the aqueous eluent during the HPLC analysis.[21] ODNs modified with lesion **1** incorporated one $^{18}O$ atom as the major species, showing a $[M-2H]^{2-}$ signal of *m/z* 917.9 and 894.9 for ODN IIa and ODN IIb, respectively. One $^{18}O$ atom incorporation was also observed in the case of oligonucleotides containing the unknown **G+135** lesion. The **Iz** containing oligonucleotides exhibited a major $[M-2H]^{2-}$ signals at *m/z* 895.5 for ODN IIa and 855.6 for ODN IIb indicating no $^{18}O$ incorporation from water in that modification.

The results of the determination of the competition of $^{16}O/^{18}O$ incorporation at different $KHSO_5$ concentrations are reported in Table III. ODNs carrying modification **1** appeared as a mixture of isomer incorporating zero to 2 atoms of $^{18}O$ depending on the reaction conditions. The $^{18}O$ incorporation on **1** was increased on decreasing the $KHSO_5$ concentration from 500 to 100 μM in both ss and ds ODN tested. Moreover, at the same $KHSO_5$ concentration, the incorporation of $^{18}O$ from water was lower on ss DNA compared to ds DNA.

These results on the origin of the oxygen atom incorporated into **1** confirmed the competition between $H_2O$ and $HSO_5^-$ for the nucleophilic attack on the guanine. Decreasing the concentration of $KHSO_5$ induced the nucleophilic attack of water to be favored (increase of $^{18}O$ content in the products of reaction). The ss DNA allowed a higher incorporation of $^{16}O$ from $KHSO_5$ due to a better accessibility of the guanines to the solvent compared to ds DNA. It is possible to observe 2 atoms of $^{18}O$ as well as 2 atoms of $^{16}O$ incorporated into **1**. Lesion **1** can thus be formed from the attack of water or $HSO_5^-$ at C5 and C8 of G. Moreover, these results gave a strong indication against the conversion of **1** into **Iz**. Under the conditions used in this work, **Iz** never showed $^{18}O$ incorporation. If **Iz** came from **1** it should reflect the isotopic pattern of its precursor. This is particularly clear at 100 μM $KHSO_5$, where 100% **Iz** appeared as $^{16}O$-labeled whereas 50% **1** was doubly $^{18}O$-labeled (Table III).

*Variation of the buffer of the reaction.* The 100% trapping of the guanine cation at C5 by $KHSO_5$ in the case of the **Iz** formation was puzzling. This data was not in accordance with the results reported in a previous paper.[21] Since the present work corresponds to a reaction performed in Tris/HCl buffer whereas in previous work labeling experiments were done in ammonium acetate buffer, we compared the incorporation of $^{18}O$ into **Iz** in different buffers. The results are summarized in Table IV. The competition between $KHSO_5$ and $H_2O$ in the access to DNA was affected by the nature of the buffer. Except in Tris/HCl buffer, **Iz** always showed an $^{18}O$ incorporation ranging from 60 to 80%. The 100% $^{16}O$-incorporation in **Iz** in Tris/HCl buffer reported in Table III, was thus an exception. The amount of $^{18}O$ incorporation in **1** was also lower in Tris/HCl buffer with respect to the other buffers.

Table IV. The $^{18}O$ incorporation in **Iz** and **1** lesions in ODN IIa during the oxidation of duplex ODN IIa/IIb in $H_2{}^{18}O$. Variation of the nature of the buffer.

| Entry | Buffer[b] | [$KHSO_5$] | Product | $^{18}O$ incorporation (%)[a] | | |
|---|---|---|---|---|---|---|
| | | | | no $^{18}O$ | 1 $^{18}O$ | 2 $^{18}O$ |
| 1 | Tris/HCl | 100 μM | **1** | - | 60 | 40 |
| 2 | | | **Iz** | 100 | - | |
| 3 | $AcONH_4$ | 100 μM | **1** | - | 40 | 60 |
| 4 | | | **Iz** | 40 | 60 | |
| 5 | $AcONEt_3H$ | 50 μM | **1** | - | 30 | 70 |
| 6 | | | **Iz** | 20 | 80 | |
| 7 | AcONa | 50 μM | **1** | - | 30 | 70 |
| 8 | | | **Iz** | 20 | 80 | |
| 9 | Phosphate | 50 μM | **1** | - | 40 | 60 |
| 10 | | | **Iz** | 30 | 70 | |

a) The values are estimated on the basis of products isotopic patterns
b) pH 7, 100 mM.

**3- Oxidized guanidinohydantoin (1) is not a precursor of Iz.** We proposed in previous papers[20,21] that **1** could be directly transformed into **Iz**, without oxidation steps (Scheme 1). If **1** was a precursor of **Iz**, it should be transformed into **Iz** in the absence of oxidizing reagent. To test it, the reaction mixtures of the oxidation of ds ODN IIa/ ODN IIb and ss ODN IIa were quenched by the addition of Hepes buffer (degradation of the excess of $KHSO_5$)[26] and incubated at 0 °C for 2 h. The HPLC profiles and the on-line ESI-MS analyses did not show any change in the products distribution over this period of time. The initial oxidation products were stable both in ds- and ss-ODNs during 2 h at 0 °C. This was an indication that dehydro-guanidinohydantoin **1** was not a precursor of **Iz** under these conditions since **Iz** formed within minutes and **1** was stable for 2 h.

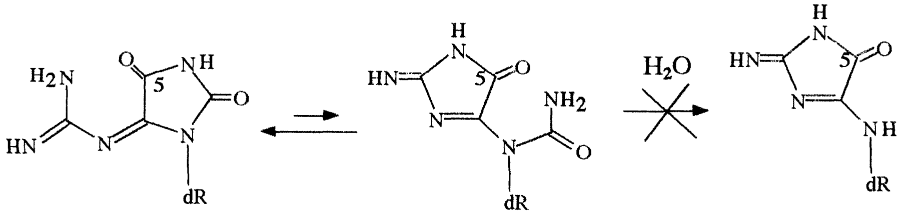

**G+4**
**(1)**

Scheme 1: Dehydro-guanidinohydantoin (**1**) lesion is not a precursor of imidazolone (**Iz**). dR stands for 2-deoxyribose or intrastrand 2-deoxyribose. 5 refers to previous C5 of G.

The oxidized DNA strand containing one **1** lesion, in the case of the oxidation of the self-complementary 5'-CAGCTG (ODN I)[21] was purified by HPLC. Just after the collect and lyophilization procedure the HPLC/ESI-MS analysis showed the almost complete degradation of **1** into two degradation products, the major one (≈ 90%) corresponded to a loss of mas of -19 amu compared to the mass of guanine and the minor one (≈ 10%) to a loss of -91 amu compared to guanine (data not shown). These two products were previously observed after a heating step (90 °C for 15 min) of the reaction medium of the oxidation of the same ds ODN I (200 mM ammonium acetate buffer pH 6.5, NaCl 100 mM) and were proposed to be 3-amino-4-carbonyl-5-[(2'-deoxy-β-D-*erythro*-pentafuranosyl)amino]-2-oxoacetic acid (oxaluric acid) derivative or **Oxa** for the major one, and urea derivative for the minor one (Scheme 2).[21] The conversion into these two products was complete after a heating step at 90 °C for 15 min in the reaction buffer, in this case the minor presence of an abasic site was also detected. The presence of imidazolone (**Iz**) or its degradation product oxazolone (**Z**)[12,13] were never detected when the isolated oligonucleotide carrying **1** lesion was subjected to lyophilisation or heating. An oligonucleotide strand modified with an **Iz** was stable under the above described conditions. A simple explanation for the formation of these two product is given in Scheme 2, **1** can lose a guanidinium moiety to give parabanic acid derivative by the hydrolysis of the imine function. It is further hydrolyzed to give the **Oxa** and urea derivatives. Parabanic acid was not detected in this work. The formation of oxaluric acid from oxidized guanidinohydantoin was also reported recently in the case of the oxidation of 8-oxo-G with singlet oxygen.[29]

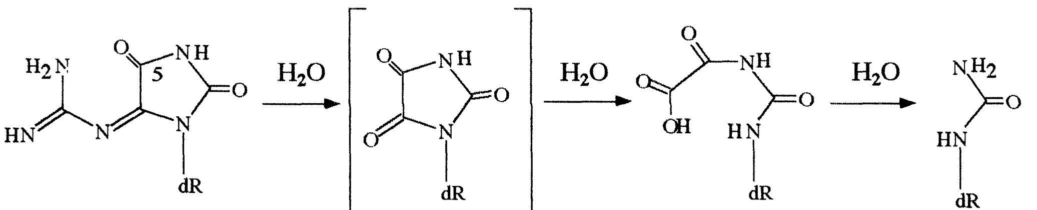

Scheme 2: degradation products of **1** in water upon heating at 90 °C (or during lyophilisation procedure). dR stands for 2-deoxyribose or intrastrand 2-deoxyribose, 5 refers to previous C5 of G and [ ] to a non-observed compound.

In summary it is clear that imidazolone did not arise from **1** as previously proposed. Compound **1** is rather the precursor of oxaluric acid derivative (**Oxa**) upon heating. Thus the oxidized guanidinohydantoin (**1**) is an independent product of guanine oxidation obtained by electron transfer.

**4- The G+34 lesion corresponds to structure 3.** To monitor the evolution of the **G+34** lesion, the modified oligonucleotide was collected from a preparative HPLC separation of an oxidation reaction performed on the self-complementary duplex 5'-CAGCTG (ODN I). The product was not very stable under the purification conditions. The analysis of the collected sample, recovered after collect and lyophilization, showed the presence of two new more polar products of degradation at Rt = 51.6 and 53.1 min besides the peak of the spiro **G+34** lesion (Rt = 55.1) (Table V, entry 1). The HPLC/ESI-MS analysis showed a $[M-2H]^{2-}$ signal at *m/z* 897.8, corresponding to a loss of 28 amu with respect to **G+34**, and thus corresponding to an increase of 6 amu compared to guanine, for the product at Rt = 51.6 min. The product at Rt = 53.1 min showed the same mass as the spiro **G+34** (*m/z* 912.0). These two products are referred to as a **G+6** and a "new **G+34**" lesions. Heating the collected sample in water (pH 5) at 90 °C for 1 h promoted the complete conversion of the spiro **G+34** into the "new **G+34**", while the **G+6** remained stable under these conditions (Table V, entry 2). On the other hand, heating the collected sample at basic pH (pH 8.8) induced the complete conversion of the spiro **G+34** into the **G+6** product. The transformation was complete within 15 min (Table V, entry 4). The "new **G+34**" and **G+6** lesion were stable for 1 h under these conditions (Table V, entry 5). Therefore, complete conversion of the the spiro **G+34** lesion into these two degradation products, the "new **G+34**" and the **G+6** lesions, was achieved by heating at 90 °C but was pH sensitive. The spiro **G+34** was transformed into a **G+6** product at pH 8.8, within 15 min whereas it transformed into a lesion of identical mass, the "new **G+34**", within 1 h upon heating at 90 °C in water (pH 5). Heating at 90 °C at pH 7 for 1 h gave an intermediate pattern of degradation of the two degradation products (Table V, entry 3). The two degradation products were stable under the different 90 °C heating conditions.

Table V. fate of the **G+34** lesion upon heating at 90 °C. Percentage of the products determined by the measurement of their HPLC peak area.

| Entry | | **G+6** 51.6[a] | **new G+34** 53.1[a] | **Spiro G+34** 55.1[a] |
|---|---|---|---|---|
| 1 | collected sample | 20 | 30 | 50 |
| 2 | 1 h, $H_2O$ | 20 | 80 | 0 |
| 3 | 1 h, pH 7[b] | 60 | 40 | 0 |
| 4 | 15 min, pH 8.8[c] | 70 | 30 | 0 |
| 5 | 1 h, pH 8.8[c] | 70 | 30 | 0 |

a) retention time (min) under HPLC conditions described in Experimental Section.
b) pH 7 = 50 mM Tris/HCl buffer, 100 mM NaCl.
c) pH 8.8 = 100 mM Tris/HCl buffer

The collected sample (Table V, entry 1) was dissolved in $H_2{}^{18}O$ Tris/HCl buffer at pH 8.8 and heated at 90 °C for 15 min. Under these conditions the spiro **G+34** transformed exclusively into the **G+6** lesion and the "new **G+34**" lesion was stable (see Table V, entry 4). The HPLC/ESI-MS analysis of the medium after 15 min heating showed that the **G+6** compound and the "new **G+34**" were unlabeled. This fact indicates that the **G+6** and the "new **G+34**" do not exchange their oxygen atoms with water. This data should be compared with the spiro **G+34** lesion that incorporated two $^{18}O$ atoms when the oxidation reaction was performed in $H_2{}^{18}O$ (Table I) and is known to exchange one of its two oxygen atoms with water (probably the oxygen atom at C8, see Scheme 3).[21] When the **G+6** lesion was formed from the doubly-labeled spiro **G+34**, it retained one $^{18}O$-atom.[21]

From literature data, **2** could undergo a rearrangement to the more stable spiro compound **3** (Scheme 3).[16, 23-25] The results on the stability of the **G+34** lesion at high temperature seem to be more in accordance with a spiro structure **3** rather than with structure **2** previously proposed. The direct degradation of **3** into the **G+6** could be the result of the attack of a molecule of water at the former C8 of G followed by the loss of $CO_2$ from the same position. The proposed structure for the **G+6** lesion is shown in Scheme 3. This structure is in agreement with labeling data. The water attack at C8 should be favored at high pH. This is in agreement with the major formation of **G+6** lesion at pH 8.8.

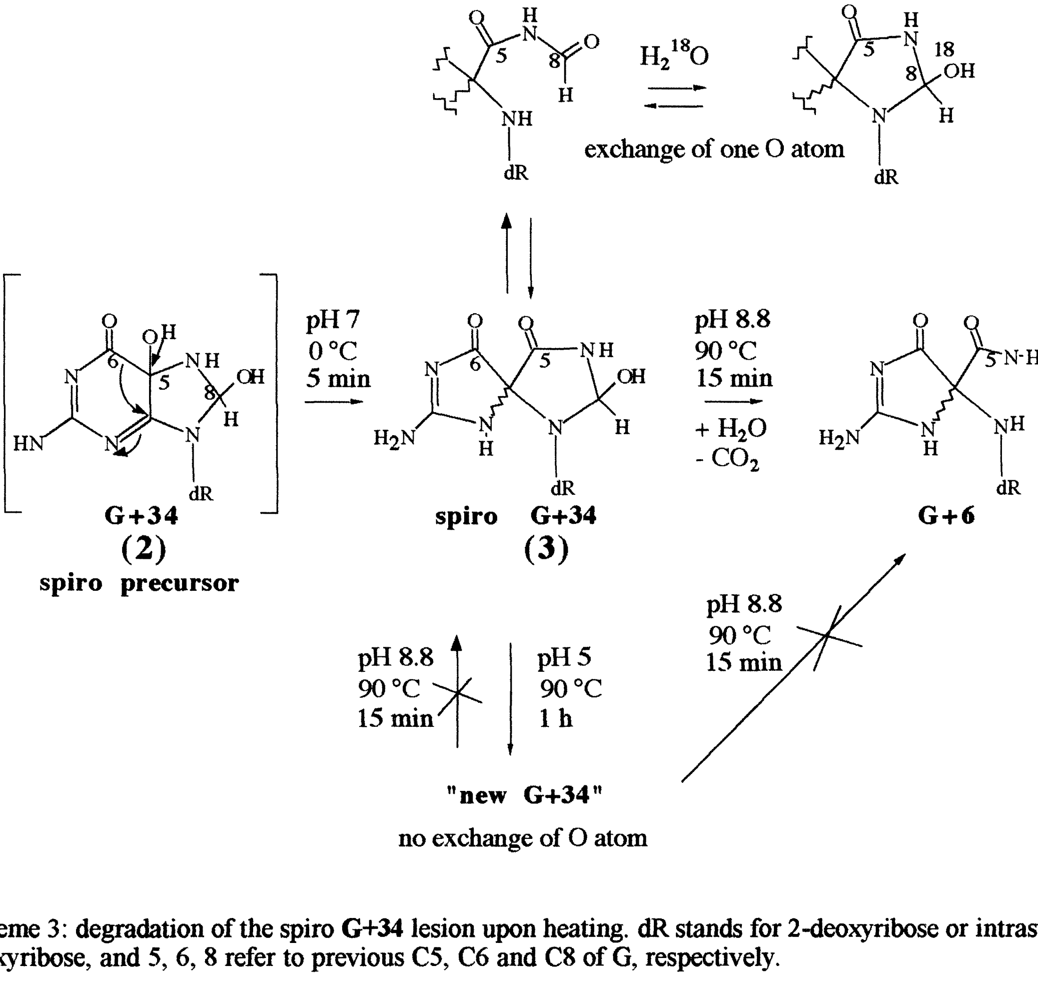

Scheme 3: degradation of the spiro **G+34** lesion upon heating. dR stands for 2-deoxyribose or intrastrand 2-deoxyribose, and 5, 6, 8 refer to previous C5, C6 and C8 of G, respectively.

The "new **G+34**" structure is not interpreted. The two **G+34** lesions (spiro and new **G+34**) are not diastereoisomers since their chemical properties are different and they are not in equilibrium. It was not possible to transform the "new G+34" lesion back to the initial one by raising the pH to 8.8. The "new **G+34**" was stable upon heating at any pH, the spiro **G+34** was unstable. The "new **G+34**" lesion did not transform into the **G+6** lesion on the contrary to the spiro **G+34** lesion. The spiro **G+34** exchanged one oxygen atom with solvent. This property is to be related to its ability to be sensitive to nucleophilic attack at C8 to transform into the **G+6** lesion. The "new **G+34**" does not exchange oxygen atoms with water when incubated in $H_2{}^{18}O$.

The so-called **G+34** lesion, observed during guanine oxidation by Mn-TMPyP/$KHSO_5$, corresponds probably to structure **3** instead of structure **2** initially proposed. If the **G+34** lesion is the spiro compound **3**, then of course it cannot be a precursor of **Iz**, but is formed via an independent reaction pathway.

### CONCLUSION

The cationic metalloporphyrin, Mn-TMPyP associated to $KHSO_5$ was used as a model reagent for the oxidation of guanine by a two-electron process. The oxidation of double-stranded oligonucleotides by Mn-TMPyP/$KHSO_5$ led to three main guanine oxidation products, namely, oxidized guanidinohydantoin (**1**), the proposed spiro compound (**3**) derivative and imidazolone lesion (**Iz**). It was shown that these three compounds are formed by independent routes. They arise from a common precursor, namely a guanine cation generated by two electron abstracted from the guanine base. This cation exhibits two electrophilic centers (C5 and C8 carbons of guanine) that may be quenched by various nucleophiles. Depending on what nucleophile ($H_2O$ or $HSO_5^-$) react at the two electrophilic carbons of guanine, the products are different. Furthermore, the primary oxidation products, oxidized guanidinohydantoin (**1**), the proposed spiro compound (**3**) derivative and imidazolone lesion (**Iz**), are susceptible to further degradation. This illustrates the high complexity of the analysis of oxidative DNA damage products.

### ACKNOWLEDGMENTS

Pr Jean Bernadou (LCC-CNRS, Toulouse) and Pr Cynthia Burrows (University of Utah) are acknowledged for fruitful discussions. A. L. is deeply indebted to the "Ministère des Affaires Etrangères", France, for a fellowship. All HPLC/ESI-MS data were obtained from the "Service de Spectrométrie de Masse de l'Université Paul Sabatier FR14-LCC-CNRS" operated by S. Richelme and C. Claparols.
Abbreviations, ss, single-stranded; ds, double-stranded; ODN, oligonucleotide; Rt, retention time; G, guanine

### REFERENCES

1. von Sonntag C. *The Chemical Basis of Radiation Biology;* Taylor and Francis, London, 1987.
2. Cadet, J., Berger, M., Douki, T., Ravanat, J.-L. *Rev. Physiol. Biochem. Pharmacol.* (1997) **131**, 1-87.
3. Steenken, S., Jovanovic, S. V. *J. Am. Chem. Soc.* (1997) **119**, 617-618.
4. Douki, T., Cadet, J. *Int. J. Radiat. Biol.* (1999) **75**, 571-581.
5. Cadet, J., Delatour, T., Douki, T., Gasparutto, D., Pouget, J.-P., Ravanat, J.-L., Sauvaigo, S. *Mutat. Res.* (1999) **424**, 9-21.
6. Sheu, C., Foote, C. S. *J. Am. Chem. Soc.* (1995) **117**, 6439-6442.
7. Kasai, H., Yamaizumi, Z., Berger, M., Cadet, J. *J. Am. Chem. Soc.* (1992) **114**, 9692-9694.
8. Cullis, P. M., Malone, M. E., Merson-Davies, L. A. *J. Am. Chem. Soc.* (1996) **118**, 2775-2781.
9. Kino, K., Saito, I., Sugiyama, H. *J. Am. Chem. Soc.* (1998) **120**, 7373-7374.
10. Gasparutto, D., Ravanat, J.-L., Gérot, O., Cadet, J. *J. Am. Chem. Soc.* (1998) **120**, 10283-10286.
11. Imidazolone nucleoside derivative (dIz) corresponds to 2-amino-5-[(2-deoxy-$\beta$ -D-*erythro*-pentofuranosyl)amino]-4*H*-imidazol-4-one and oxazolone nucleoside derivative (dZ) to 2,2-diamino-4-[2-deoxy-$\beta$-D-*erythro*pentofuranosyl)amino]-2,5-dihydrooxazol-5-one.
12. Cadet, J., Berger, M., Buchko, G. W., Joshi, P. C., Raoul, S., Ravanat, J.-L. *J. Am. Chem. Soc.* (1994) **116**, 7403-7404.
13. Raoul, S., Berger, M., Buchko G. W., Joshi, P. C., Morin, B., Weinfeld, M., Cadet, J. *J. Chem. Soc., Perkin Trans. 2*, (1996) **2**, 371-381.
14. Prat, F., Houk, K. H., Foote, C. S. *J. Am. Chem. Soc.* (1998) **120**, 845-846.
15. Burrows, C. J., Muller, J. G. *Chem. Rev.* (1998) **98**, 1109-1151.
16. Luo, W., Muller, J. G., Rachlin, E. M., Burrows, C. J., *Organic Lett.* (2000) **2**, 613-616.
17. Niles, J. C., Burney, S., Singh, S. P., Wishnok, J. S., Tannenbaum, S. R. *Proc. Natl. Acad. Sci. USA* (1999) **96**, 11729-11734.
18. Niles, J. C., Wishnok, J. S., Tannenbaum, S. R. *Chem. Res. Toxicol.* (2000) **13**, 390-396.
19. Mn-TMPyP stands for manganese(III)-bis-aqua-*meso*-tetrakis(4-*N*-methylpyridiniumyl)-porphyrin.
20. Vialas, C., Pratviel, G., Claparols, C., Meunier, B. *J. Am. Chem. Soc.* (1998) **120**, 11548-11553.
21. Vialas, C., Claparols, C., Pratviel, G., Meunier, B. *J. Am. Chem. Soc.* (2000) **122**, 2157-2167.
22. Chworos, A., Coppel, Y., Dubey, I., Pratviel, G., Meunier, B. submitted.
23. Shibutani, S., Gentles, G., Iden, C. R., Johnson, F., *J. Am. Chem. Soc.* (1990) **112**, 5667-5668.
24. Johnson, F., Huang, C.-Y., Yu, P.-L. *Environmental Health Perspectives* (1994) **102**, 143-149.
25. Modric, N., Poje, M., Gojmerac-Jvsic A. *Biorg. Med. Chem. Lett.* (1994) **4**, 1685-1686.
26. Bernadou, J., Pratviel, G., Bennis, F., Girardet, M., Meunier, B. *Biochemistry* (1989) **28**, 7268-7275.
27. McLuckey, S. A., Habibi-Goudarzi, S. *J. Am. Chem. Soc.* (1993) **115**, 12085-12095.
28. Vialas, C., Pratviel, G., Meyer, A., Rayner, B., Meunier, B. *J. Chem. Soc., Perkin Trans 1* (1999) 1201-1205.
29. Duarte, V., Gasparutto, D., Yamaguchi, L. F., Ravanat, J.-L., Martinez, G. R., Medeiros, M. H. G., Di Mascio, P., Cadet, J. *J. Am. Chem. Soc.* (2000) **122**, 12622-12628.

